# Point-of-care Ultrasound in Morbidity and Mortality Cases in Emergency Medicine: Who Benefits the Most?

**DOI:** 10.5811/westjem.2020.7.47486

**Published:** 2020-10-28

**Authors:** Andrew J. Goldsmith, Hamid Shokoohi, Michael Loesche, Ravish C. Patel, Heidi Kimberly, Andrew Liteplo

**Affiliations:** * Harvard Medical School, Department, Boston, Massachusetts; † Brigham and Women’s Hospital, Department of Emergency Medicine, Boston, Massachusetts; ‡ Massachusetts General Hospital, Department of Emergency Medicine, Boston, Massachusetts; § Medical College of Georgia School of Medicine, Department, Augusta, Georgia

## Abstract

**Introduction:**

Point-of-care ultrasound (POCUS) is an essential tool in the timely evaluation of an undifferentiated patient in the emergency department (ED). Our primary objective in this study was to determine the perceived impact of POCUS in high-risk cases presented at emergency medicine (EM) morbidity and mortality (M&M) conferences. Additionally, we sought to identify in which types of patients POCUS might be most useful, and which POCUS applications were considered to be highest yield.

**Methods:**

This was a retrospective survey of cases submitted to M&M at an EM residency program that spans two academic EDs, over one academic year. Postgraduate year 4 (PGY) residents who presented M&M cases at departmental sessions were surveyed on perceived impacts of POCUS on individual patient outcomes. We evaluated POCUS use and indications while the POCUS was used.

**Results:**

Over the 12-month period, we reviewed 667 cases from 18 M&M sessions by 15 PGY-4 residents and a supervising EM attending physician who chairs the M&M committee. Of these cases, 75 were selected by the M&M committee for review and presentation. POCUS was used in 27% (20/75) of the cases and not used in 73% (55/75). In cases where POCUS was not used, retrospective review determined that if POCUS had been used it would have “likely prevented the M&M” in 45% (25/55). Of these 25 cases, the majority of POCUS applications that could have helped were cardiac (32%, 8/25) and lung (32%, 8/25) ultrasound. POCUS was felt to have greatest potential in identifying missed diagnoses (92%, 23/25), and decreasing the time to diagnosis (92%, 23/25). Patients with cardiopulmonary chief complaints and abnormal vital signs were most likely to benefit. There were seven cases (35%, 7/20, 95% CI 15–59%) in which POCUS was performed and thought to have possibly adversely affected the outcome of the M&M.

**Conclusion:**

POCUS was felt to have the potential to reduce or prevent M&M in 45% of cases in which it was not used. Cardiac and lung POCUS were among the most useful applications, especially in patients with cardiopulmonary complaints and in those with abnormal vital signs.

## INTRODUCTION

Medical errors have been reported to be the third leading cause of death in the United States.^1^ Specifically, diagnostic errors account for an estimated 40,000–80,000 annual deaths in this country.^2^ In critical care patients this is further exemplified as one study showed that upwards of 10% of intensive care unit (ICU) patients had lethal misdiagnoses on autopsy.^3^ Diagnostic errors are under-reported and underemphasized; this is an understudied area of patient-safety that can affect the well-being of providers involved with the errors.^4^

Point-of-care ultrasound (POCUS) is an essential tool in the timely evaluation of critically ill patients and those with undifferentiated diagnoses. For this reason, POCUS training is a growing part of medical education, particularly in emergency medicine (EM) where accreditation training requirements exist, and residents are required by the Accreditation Council for Graduate Medical Education to demonstrate POCUS competency.^4^ Additionally, the American College of Emergency Physicians has released a policy statement including guidelines and recommendations for POCUS education for emergency physicians.^5^ Successful implementation of POCUS requires emergency physicians to acquire and interpret images, as well as apply and integrate these interpretations into clinical practice.

There is an ever-growing body of literature describing the diagnostic utility of POCUS for specific diseases.^6^–^8^ Further, there is extensive research describing how experienced practitioners can improve diagnostic certainty in undifferentiated hypotensive patients.^9^ For example, in hypotensive trauma patients, a positive focused assessment with sonography in trauma (FAST) exam in the ED has shown to decrease time to the operating room and length of stay with very high specificity.^10^ Also, POCUS evaluation of patients with acute dyspnea has shown to reduce diagnostic time with good concordance with admission diagnosis.^11^ In the ED, POCUS plays an increasingly important role in a patient’s ultimate timely diagnosis and thereby treatment.^6^,^10^–^12^ This has led practitioners to believe that POCUS may improve patient outcomes.

Departmental morbidity and mortality (M&M) conferences are routinely held to investigate individual and systematic errors that contribute to preventable medical errors that lead to patient morbidity and mortality. M&M review has been used in the past to draw meaningful data about preventable deaths and trends in the care of these patients.^13^ In this paper, we use similar methodology to review M&M cases for the purpose of assessing the impact that POCUS might have on patient outcomes.

Our primary goal was to determine the perceived role of POCUS on affecting clinical outcomes on M&M cases by performing a descriptive analysis of the use of POCUS in cases reviewed for M&M. We also sought to determine which POCUS applications and in which types of patients ultrasound had the most perceived value. Having this information could guide emergency physicians as to what POCUS to perform and in whom. Our goal was to improve patient care by sharing and examining our collective experiences in high-yield M&M cases for using POCUS. Despite recognition that clinical integration is essential, there is limited published data on actual patterns of usage of POCUS by emergency physicians. To our knowledge no study has examined the potential role of POCUS on cases reviewed in two emergency departments’ (ED) M&M conferences.

## METHODS

### Study Setting and Population

This retrospective study was done at two large academic EDs with annual volumes of 120,000 and 70,000 patients. Both institutions have an emergency ultrasound (US) division, emergency US fellowship program, and share a four-year EM residency training program with 60 EM residents postgraduate years 1–4 (PGY). This study was reviewed by the institutional review board and determined to be exempt.

**Population Health Research Capsule**

What do we already know about this issue?

*Point-of-care ultrasound (POCUS) is an essential tool in the timely evaluation of an undifferentiated patient in the emergency department (ED)*.

What was the research question?

*The objective was to determine the perceived impact of POCUS in high-risk cases presented at morbidity and mortality (M&M) conferences*.

What was the major finding of the study?

*POCUS has the potential to reduce or prevent M&M in 45% of cases in which it was not used*.

How does this improve population health?

*As diagnostic errors account for an estimated 40,000–80,000 annual deaths in the United States, POCUS may help reduce this within the ED*.

### Selection of Participants

Cases were reviewed monthly in the departmental M&M conference as part of routine departmental quality assurance. PGY-4 EM residents prepared M&M cases for review with a faculty EM attending physician as part of this process, and not for research purposes. All PGY-4 EM residents were asked to participate in the study survey. Participation was voluntary. There were no exclusion criteria.

### Study Design

All ED cases were subject to review from July 2018–June 2019. All cases that resulted in a death in the ED, all deaths within 24 hours of an ED encounter, and all upgrades to an ICU within 24 hours were automatically reviewed for possible clinical or system errors. In addition, any cases referred by nursing, ED providers, and providers from other departments were reviewed.

For each M&M session, a PGY-4 resident was provided a list of all cases for review over a designated time period. This resulted in approximately 60–75 cases over about a 40-day period. Each M&M conference was specific to one hospital. All information was obtained by retrospective chart review. After reviewing each case, the PGY-4 resident submitted a summary of each case to a faculty mentor, an attending physician responsible for departmental M&M review. Together, the PGY-4 resident and attending physician identified all cases that were considered to have potential patient care concerns while in the ED. This review was done as routine departmental quality assurance and not for purposes of the study.

After all cases were reviewed, a study investigator surveyed the PGY-4 residents about all of the cases in which there were possible concerns about patient care as determined by the PGY-4 EM resident and an EM attending. The survey addressed questions regarding the use of POCUS in M&M cases. Specifically, the resident was asked:

- In cases when POCUS was performed, did POCUS contribute to the M&M?
- In cases when POCUS was not performed, would it likely have prevented the M&M if it had been done?
- If so, which application(s) would have helped, and how?

Residents were instructed that when assessing the potential of POCUS to prevent M&M, they should assume that POCUS would have been appropriately performed, interpreted, and integrated. In addition, we collected information regarding patients’ initial chief complaints and initial triage vital signs. An US fellow and/or an US fellowship-trained EM attending administered the survey. Verbal consent was obtained for all participants. The PGY-4 resident was blinded to the purposes of the study. An EM attending with fellowship training in POCUS was then presented with the same cases. The attending, blinded to the resident’s assessment, was then asked the same questions. Additionally, the attending was asked specifically if he or she would have performed a POCUS if presented with the same clinical case and timeline.

We collected and managed study data using REDCap (Vanderbilt University, Nashville, TN) electronic data capture tools hosted at Massachusetts General Hospital.^14^,^15^ The same software stored and de-identified all demographic and clinical data obtained.

### Data Analysis

All data obtained was de-identified, exported to, and analyzed in Microsoft Office 365 Excel (Microsoft Corporation, Redmond, WA). We used descriptive statistical analysis to compare the data. We calculated the overall percentage of cases where M&M may have been affected by POCUS, as assessed by a PGY-4 resident. We then performed subgroup analyses stratified by chief complaint, vital signs, type of POCUS, and how POCUS may have affected M&M. A kappa value was then calculated for interobserver agreement between the PGY-4 residents and the US EM attending. We calculated proportion confidence intervals (CI) of 95% using our sample sizes.

## RESULTS

Between the two academic hospitals, there were a total of 18 M&M conferences (nine per each hospital) over the 12-month period. These were reviewed by 15 different PGY-4 residents; three residents reviewed cases for two different conferences. There was a 100% response rate among residents.

**Figure 1:**
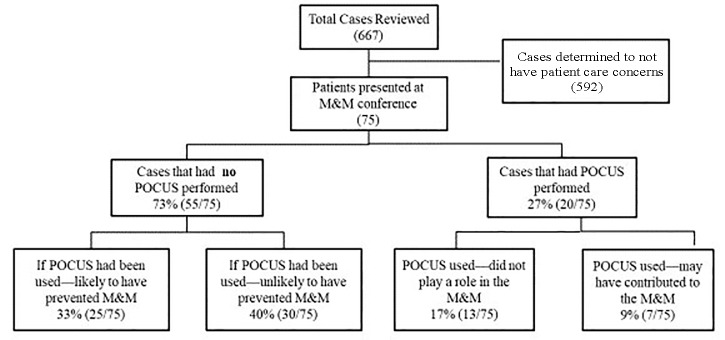
Flow diagram of morbidity and mortality cases. *POCUS*, point-of-care ultrasound; *M&M*, morbidity and mortality.

Of the 667 cases reviewed 75 cases were determined to have patient care concerns. POCUS was used in 27% (20/75, 95% CI, 17–38%) and not used in 73% (55/75, 95% CI, 62–83%) (Figure 1). In cases where POCUS was not used, retrospective review determined that if POCUS had been used it would have “likely prevented the M&M” in 45% (25/55, 95% CI, 32–59%) There was a kappa value of 0.85 between the PGY-4 residents and the fellowship-trained EM attending in making this assessment. The US EM attending would have clinically used POCUS in 52% (13/25, 95% CI, 31%–72%) of these cases.

The most common chief complaints were shortness of breath 23% (17/75), trauma 15% (11/75), and cardiac arrest 12% (9/75) (Table 1). Thirty-six percent (27/75) were deaths within the ED. Of the 45% (25/55) of cases in which POCUS was not used but was felt would have likely prevented the M&M, the most common presentations were chest pain (75%, 6/8), shortness of breath (47%, 8/17), and trauma (36%, 4/11).The most common vital sign abnormalities were tachycardia 49% (37/75) and hypoxia 26% (20/75). Of the cases with these abnormalities, POCUS was felt likely to have made an impact if it had been used in 40% (8/20, 95% CI, 19–64%), of the hypoxic cases and 30% (11/37, 95% CI, 16–47%), of the tachycardic cases.

**Figure 2:**
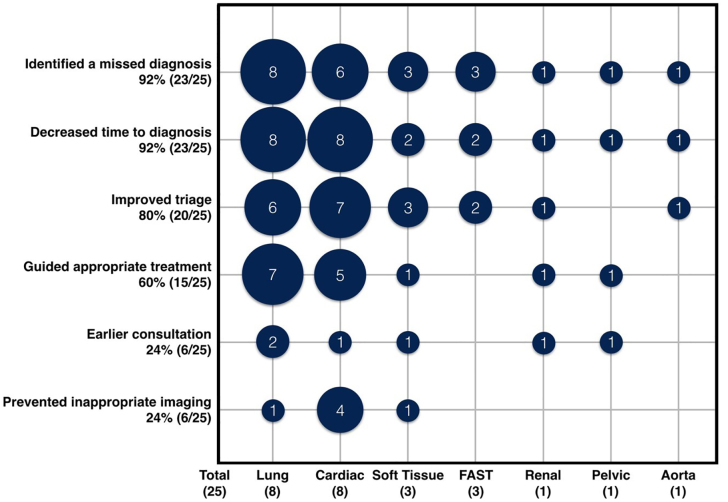
Perceived impact of point-of-care ultrasound: applications versus mechanism by which POCUS may have reduced or prevented morbidity ad mortality (N = 25 cases, multiple mechanisms per case were possible). *FAST*, focused assessment with sonography in trauma.

The perceived benefit of POCUS in preventing M&M was varied. POCUS often had the potential to have improved care by multiple different mechanisms. Mechanisms by which POCUS might have prevented the M&M were as follows: identified a missed diagnosis (92%, 23/25, 95% CI, 74–99%); decreased time to diagnosis (92%, 23/25, 95%, CI 74–99%); improved triage to an area of higher level of care (80%, 20/25, 95% CI, 59–93%); guided appropriate treatment (60%, 15/25, 95% CI, 39–79%); earlier consultation (24%, 6/25, 95% CI, 9–45%); and prevented inappropriate imaging (24%, 6/25, 95% CI, 9–45%). The POCUS applications that would have helped the most were cardiac (32%, 8/25, 95% CI, 15–54%), and lung (32%, 8/25, 95% CI 15–54%). This data is summarized in Figure 2.

There were seven cases (35%, 7/20, 95% CI 15–59%) in which POCUS was performed and thought to have possibly adversely affected the outcome of the M&M. The cases were classified by type to characterize the errors. Of these errors, in four POCUS was incorrectly integrated into clinical care, in two POCUS was incorrectly performed, and in two POCUS was incorrectly interpreted. These cases are summarized in Table 2.

## DISCUSSION

An aggregate review of M&Ms over a one-year period showed the perceived potential for POCUS to prevent M&M. This is the first report of which we are aware that examines POCUS through a hospital’s M&M conference. In this pool of high-yield cases we determined that in up to 33% (25/75) of cases of M&M, POCUS had not been done but might have helped to prevent the M&M. Of course, POCUS findings would be only one of many needed pieces of information that could have changed management, identified diagnoses, or decreased time to diagnoses.

Whether or not POCUS would have been done is harder to assess. An EM attending with US training stated that based on the retrospective information about the case, he would have personally performed a POCUS in only 52% (13/25) of cases. It should be noted that an US EM attending’s usage is likely to be higher than that of an EM attending without specialized US training; thus, this number may be an overestimation. In the rest of the cases where it was felt that POCUS might have prevented M&M, the US EM attending did not think that he would have performed a POCUS. For many of the cases, the US findings might have been considered to be advanced (ie, endocarditis, focal wall-motion abnormalities) and probably fell outside the scope of standard POCUS in EM. As emergency physicians become more and more facile with POCUS, it is possible that these applications may become more commonplace.

In this study, M&M was used as a surrogate of critically ill patients with significant adverse outcomes as it has been identified in previous literature within EM.^16^,^17^ Our data speak to the importance of POCUS use in the routine care of patients while in the ED, especially in those who are critically ill.

One of the most difficult aspects of POCUS utilization is knowing in which patients to use it. Even when an emergency physician has competence in performing, interpreting, and integrating US, if it is not done then there is no benefit to the patient. Having greater diagnostic accuracy earlier in a patient’s work-up could potentially allow for optimization of care during the golden hour with streamlined treatment, better decision-making about imaging, earlier consultation, and more accurate disposition. However, POCUS takes time and so performing it in every patient may not be an efficient use of ED resources or physician time. Our results showed that patients with chief complaints of chest pain, shortness of breath, and trauma made up approximately 80% of the M&Ms where POCUS was thought to be able to help prevent its outcome. This is not surprising as chief complaints of chest pain and shortness of breath comprise a large number of ED visits and are often caused by diagnoses with high mortality.^18^ Our data also show that vital sign abnormalities were common in M&M cases where POCUS may have made a difference. Specifically, patients who were tachycardic and/or hypoxic were the most likely to benefit from POCUS. This information can be used to guide physician decision-making with critically ill patients and clinical protocols in EDs.

Additionally, these data can inform ultrasound education in EM residencies and support the idea of advocating for “POCUS first” algorithms in patients presenting with chest pain, shortness of breath, hypoxia, and/or tachycardia. As FAST has been integrated into the Advanced Trauma Life Support algorithm for trauma patients, cardiac POCUS is starting to be incorporated into Advanced Cardiac Life Support for routine cardiac arrest care in the ED.^19^ It may be reasonable to develop similar algorithms for patients with hypoxia and/or tachycardia or with chief complaints of chest pain and shortness of breath with the intent of improving patient outcomes. Although it is not reasonable for all patients, highly targeted POCUS for a specific patient population with cardiopulmonary complaints is reasonable. Further, this may help educators teach trainees which patients clinically may have the highest benefit of a POCUS when clinicians must triage multiple sick patients at once. A few studies have attempted to describe their integration;^12^,^20^ however, further research is needed on specific patient outcomes.

Of the 75 cases that were presented to M&M, in 9% (7/75) POCUS may have been one component that negatively impacted the case. To inform educational endeavors, we analyzed the results by the three components of POCUS: 1) performing the POCUS and acquiring images; 2) interpreting the images; and 3) integrating the findings into clinical care. In half of the errors, the POCUS was both done and interpreted correctly, but the integration of clinical findings was flawed. This knowledge has important implications on POCUS education. POCUS curricula in EM residencies are comprised largely of scan shifts in which acquisition and interpretation of images are heavily emphasized, but integration of findings may not be. These data highlight the importance of also focusing integration of POCUS findings into clinical care needs and emphasize the need for comprehensive POCUS training.

In a quarter of the errors, POCUS was incorrectly performed. Both of the cases were related to procedural guidance. It is not entirely clear how POCUS was or was not involved in these cases as we did not perform image review, but this does speak to the importance of skills training, perhaps in simulation settings. Physicians from non-EM specialties were involved in some of these procedural errors and highlights the need for POCUS education to all services who care for patients in the ED. Finally, interpretation of POCUS was the issue in a quarter of the errors. In both of these cases (necrotizing fasciitis and focal cardiac tamponade), findings extended beyond the traditional questions that POCUS answers. This highlights a vulnerability of POCUS, in that even in these cases we as providers are responsible for images that we acquire and their findings. Identifying examples of these vulnerabilities through review of M&M cases can be one tool that we as educators use to further the education of our physicians. Although POCUS was involved in 9% of adverse cases associated with M&M, it does not suggest that US in and of itself is a dangerous tool. Rather, it underscores the importance of competence in using US and the need for high quality and continuing training.

The notion of POCUS identifying hard-to-make diagnoses is also supported by our study. Mechanisms of how POCUS was perceived to help prevent M&M were noted and quantified. POCUS was perceived to be most potentially useful in its ability to identify missed diagnoses (92% of cases) and decrease the time to diagnosis (92%). Given this ability, the threshold for performance of US in all patients with a questionable diagnosis should be very low. Ultimately, our study supports the idea that US may have a role in decreasing diagnostic and procedural errors, thereby improving patient care. However, it also shows that if US is done it needs to be done well, accurately, and integrated into patient care correctly.

## LIMITATIONS

There are several limitations in our study. One limitation was response bias as the results represent the views of the individual respondents. All cases were initially reviewed for patient-related concerns by both the PGY-4 and an EM attending who were not a part of the study, so selection of cases was not biased. However, our surveys were completed by PGY-4 residents only and represent their views of the case. By using PGY-4s it is reasonable to say that they are not expert users of POCUS or experts in medical management, leading to possible inaccurate results. However, an US-trained attending reviewed all the cases and had high agreement with the PGY-4 opinions of the case. Second, all cases that had possible clinical errors were reviewed by an EM attending for agreement.

Another limitation was that the individuals administrating the survey were US faculty. This could have potentially led to some indirect bias on the part of the PGY-4s’ responses. Our study was also limited in that we had a relatively low sample size and it was done in academic EDs, potentially leading to limitations regarding the generalizability of our study. However, past studies surrounding M&M process have had similar numbers when reporting.^17^,^18^ Finally, perfect conditions were assumed in cases where POCUS was felt to potentially have a role in preventing an M&M. In reality, it is not the case that images are always correctly obtained, interpreted, and integrated; so the perceived potential benefit of POCUS is a theoretical one. There are many factors related to the patient, provider, and clinical environment that also affect the utility of POCUS and likelihood that it is performed that we could not control for in our model.

## CONCLUSION

In our study, the use of POCUS could potentially have positively impacted 33% of departmental M&M cases in which there were concerns about patient care. POCUS would be most likely to prevent M&M in patients with chest pain, shortness of breath, trauma, tachycardia, or hypoxia. Cardiac and lung ultrasound were the applications felt to have the greatest potential to minimize M&M. Clinical integration is an essential component of POCUS competency, and it should be prioritized and taught in appropriate platforms. This information can be useful in guiding POCUS educational curricula and clinical decision-making. A prospective study is needed to determine the actual impact of POCUS on patient-centered outcomes in high-risk patients in the ED.

## Figures and Tables

**Table 1:** Chief complaints or reasons for referral and vital signs of morbidity and mortality cases reviewed (N = 75).

	POCUS may have prevented M&M	Total cases (N = 75)
Chief complaint		
Chest pain	75% (6/8)	11% (8/75)
Procedural complication	67% (2/3)	4% (3/75)
Shortness of breath	47% (8/17)	23% (17/75)
Trauma	36% (4/11)	15% (11/75)
Altered mental status	29% (2/7)	9% (7/75)
Cardiac arrest	22% (2/9)	12% (9/75)
Abdominal pain	17% (1/6)	8% (6/75)
Other	0% (0/8)	11% (8/75)
Headache	0% (0/4)	5% (4/75)
Medication error	0% (0/3)	3% (2/75)
Vital signs		
Hypoxic	40% (8/20)	26% (20/75)
Tachycardic	30% (11/37)	49% (37/75)
Febrile	29% (2/7)	9% (7/75)
Hypotensive	26% (5/19)	25% (19/75)

*POCUS*, point-of-care ultrasound; *M&M*, morbidity and mortality.

**Table 2:** Description of cases that POCUS may have contributed to the M&M.

Case	Case description	Ultrasound contribution	Type of error		

			Incorrectly interpreted	Incorrectly performed	Incorrectly integrated
1	Possible septic shock with acute on chronic RV failure.	Severe RV dysfunction correctly identified, however 4L of IVF given causing fluid overload.			X
2	Hemothorax. Liver injury occurred during chest tube placement.	Hemothorax correctly identified but ultrasound not used to guide chest tube placement.		X	X
3	Persistent tachycardia. PE not considered.	RV dilatation correctly identified but not incorporated into care.			X
4	Hemothorax after ultrasound-guided ipsilateral central line placement.	Presumed vascular injury secondary to central venous access attempt. Unclear how procedure was done.		X	
5	Trauma with hypotension.	+FAST correctly identified. No surgery consults until after CT.			X
6	Leg infection treated as cellulitis as outpatient. Returned with necrotizing fasciitis.	Ultrasound correctly identified soft tissue edema, but providers missed subcutaneous air, which was visible.	X		
7	Shortness of breath. Pleural and pericardial effusions identified, admitted.	Pericardial effusion correctly identified, but not read as early tamponade delaying emergent consults.	X		
Total (8 errors/7cases)			25% (2/8)	25% (2/8)	50% (4/8)

*M&M*, morbidity and mortality; *RV*, right ventricle; *IVF*, intravenous fluid; *PE*, pulmonary embolism; *FAST*, focused assessment with sonography in trauma; *CT*, computed tomography.

## References

[1] Makary MA, Daniel M (2016). Medical error: the third leading cause of death in the US. BMJ.

[2] Newman-Toker DE, Pronovost PJ (2009). Diagnostic errors: the next frontier for patient safety. JAMA.

[3] Winters B, Custer J, Galvagno SM (2012). Diagnostic errors in the intensive care unit: a systematic review of autopsy studies. BMJ Qual Saf.

[4] Kaur AP, Levinson AT, Monteiro JFG (2019). The impact of errors on healthcare professionals in the critical care setting. J Crit Care.

[5] American College of Emergency Physicians (2016). Ultrasound Guidelines: Emergency, Point-of-Care and Clinical Ultrasound Guidelines in Medicine.

[6] Rempell JS, Noble VE (2011). Using lung ultrasound to differentiate patients in acute dyspnea in the prehospital emergency setting. Crit Care.

[7] Mallin M, Craven P, Ockerse P (2015). Diagnosis of appendicitis by bedside ultrasound in the ED. Am J Emerg Med.

[8] Shokoohi H, Boniface KS, Pourmand A (2015). Bedside ultrasound reduces diagnostic uncertainty and guides resuscitation in patients with undifferentiated hypotension. Crit Care Med.

[9] Seif D, Perera P, Mailhot T (2012). Bedside ultrasound in resuscitation and the rapid ultrasound in shock protocol. Crit Care Res Pract.

[10] Melniker LA, Leibner E, McKenney MG (2006). Randomized controlled clinical trial of point-of-care, limited ultrasonography for trauma in the emergency department: the first sonography outcomes assessment program trial. Ann Emerg Med.

[11] Zanobetti M, Scorpiniti M, Gigli C (2017). Point-of-care ultrasonography for evaluation of acute dyspnea in the ED. Chest.

[12] Liteplo AS, Marill KA, Villen T (2009). Emergency thoracic ultrasound in the differentiation of the etiology of shortness of breath (ETUDES): sonographic B-lines and N-terminal pro-brain-type natriuretic peptide in diagnosing congestive heart failure. Acad Emerg Med.

[13] Schoeneberg C, Schilling M, Probst T (2014). Preventable and potentially preventable deaths in severely injured elderly patients: a single-center retrospective data analysis of a German trauma center. World J Surg.

[14] Harris PA, Taylor R, Thielke R (2009). Research Electronic Data Capture (REDCap) - A metadata-driven methodology and workflow process for providing translational research informatics support. J Biomed Inform.

[15] Harris PA, Taylor R, Minor BL (2019). The REDCap consortium: building an international community of software platform partners. J Biomed Inform.

[16] Aaronson EL, Wittels KA, Nadel ES (2015). Morbidity and mortality conference in emergency medicine residencies and the culture of safety. West J Emerg Med.

[17] Seigel TA, McGillicuddy DC, Barkin AZ (2010). Morbidity and mortality conference in emergency medicine. J Emerg Med.

[18] Niska R, Bhuiya F, Xu J (2010). National Hospital Ambulatory Medical Care Survey: 2007 emergency department summary. Natl Health Stat Report.

[19] Arntfield R, Pace J, Hewak M (2016). Focused transesophageal echocardiography by emergency physicians is feasible and clinically influential: observational results from a novel ultrasound program. J Emerg Med.

[20] Perera P, Mailhot T, Riley D (2010). The RUSH exam: Rapid Ultrasound in SHock in the evaluation of the critically ill. Emerg Med Clin North Am.

